# Recommendations From a Descriptive Evaluation to Improve Screening Procedures for Web-Based Studies With Couples: Cross-Sectional Study

**DOI:** 10.2196/15079

**Published:** 2020-05-12

**Authors:** Jason W Mitchell, Tanaka M D Chavanduka, Stephen Sullivan, Rob Stephenson

**Affiliations:** 1 Office of Public Health Studies Myron B Thompson School of Social Work University of Hawai’i at Mānoa Honolulu, HI United States; 2 The Center for Sexuality and Health Disparities School of Nursing University of Michigan Ann Arbor, MI United States; 3 Department of Systems, Population and Leadership School of Nursing University of Michigan Ann Arbor, MI United States

**Keywords:** couples, methods, internet

## Abstract

**Background:**

Although there are a number of advantages to using the internet to recruit and enroll participants into Web-based research studies, these advantages hinge on data validity. In response to this concern, researchers have provided recommendations for how best to screen for fraudulent survey entries and to handle potentially invalid responses. Yet, the majority of this previous work focuses on screening (ie, verification that individual met the inclusion criteria) and validating data from 1 individual, and not from 2 people who are in a dyadic relationship with one another (eg, same-sex male couple; mother and daughter). Although many of the same data validation and screening recommendations for Web-based studies with individual participants can be used with dyads, there are differences and challenges that need to be considered.

**Objective:**

This paper aimed to describe the methods used to verify and validate couples’ relationships and data from a Web-based research study, as well as the associated lessons learned for application toward future Web-based studies involving the screening and enrollment of couples with dyadic data collection.

**Methods:**

We conducted a descriptive evaluation of the procedures and associated benchmarks (ie, decision rules) used to verify couples’ relationships and validate whether data uniquely came from each partner of the couple. Data came from a large convenience sample of same-sex male couples in the United States, who were recruited through social media venues for a Web-based, mixed methods HIV prevention research study.

**Results:**

Among the 3815 individuals who initiated eligibility screening, 1536 paired individuals (ie, data from both partners of a dyad) were assessed for relationship verification; all passed this benchmark. For data validation, 450 paired individuals (225 dyads) were identified as fraudulent and failed this benchmark, resulting in a total sample size of 1086 paired participants representing 543 same-sex male couples who were enrolled. The lessons learned from the procedures used to screen couples for this Web-based research study have led us to identify and describe four areas that warrant careful attention: (1) creation of new and replacement of certain relationship verification items, (2) identification of resources needed relative to using a manual or electronic approach for screening, (3) examination of approaches to link and identify both partners of the couple, and (4) handling of *bots*.

**Conclusions:**

The screening items and associated rules used to verify and validate couples’ relationships and data worked yet required extensive resources to implement. New or updating some items to verify a couple’s relationship may be beneficial for future studies. The procedures used to link and identify whether both partners were coupled also worked, yet they call into question whether new approaches are possible to help increase linkage, suggesting the need for further inquiry.

## Introduction

### Background

In the United States, 90% of adults use the internet for social connections and information searching [1], suggesting internet usage has become increasingly a normative behavior. More adults own a smartphone than not (83% in urban and suburban, 71% in rural areas), with a similar representation of having broadband internet at home [2]. Further, 70% of adults have and use at least one social media account, and usage—across multiple accounts—continues to increase with respect to a person’s age, race, gender, income, education, and community (ie, urban, suburban, rural) [3]. These trends equate to more and more research studies being conducted on the Web.

There are a number of advantages to conducting research studies on the Web. Compared with in-person methods, the internet enables researchers with more efficient modes (eg, targeted social media advertisements) to access small and/or hard-to-reach populations, including sexual and gender minority groups [4]. With respect to time, Web-based recruitment efforts can reach larger samples of potential research participants in shorter periods of time compared with more traditional in-person outreach methods. Use of Web-based methods to enroll and collect data from participants may also benefit researchers by shortening the amount of time needed to prepare data for use in analytic software programs.

There are, however, methodological challenges associated with conducting studies on the Web with respect to data validity [5-10], as anonymity and lack of in-person contact prohibits researchers from knowing who or what are providing data. Data validity may be a particular concern when incentives or compensation are offered. For instance, a participant may enter false information about themselves for purposes of earning the incentive (ie, misrepresentation for eligibility) [5,11-16], or enter the study multiple times to earn multiple incentives or increase the chances of earning an incentive, by either pretending to be different participants or the same individual (ie, deduplication or multiple data entry) [8,10,12,13,17]. Web-based research that lacks mechanisms to detect such instances of invalid data entries will negatively impact the study’s findings and associated recommendations.

In response to this concern, researchers have provided recommendations related to screening for fraudulent survey entries and regarding how best to handle potentially invalid responses when they do occur. One recommendation is to *use* all data by categorizing survey entries into groups—valid, suspicious, and invalid—along with accompanying pre and post hoc decisions for how best to handle the data [6]. This approach allows researchers to keep all data for analysis, assess differences between the categories of survey entries, detect whether any data entries were incorrectly categorized, and to fine-tune the pre and post doc decisions to categorize or label data entries in future studies. This process uses a less conservative approach by examining all data entries and requires more time to execute, although it may help expedite the screening process (ie, detecting invalid data) in future Web-based projects. Another recommendation is to assess survey responses for patterns, such as whether the same response was repeatedly used to answer questions (eg, always the second response option), if a consistent pattern was used to respond to questions throughout the survey (eg, acbd, acbd), and whether a participant provided the same response to the same question asked at different points in the same survey (ie, internal consistency) [4,16,18]. Another recommendation includes reviewing the internet protocol (IP) address in conjunction with other data collected from the participants, such as their state of current residence or zip code to examine whether this information concurs with one another (ie, IP address matches state) [7,13,16,19].

The majority of this previous work focuses on screening (ie, verification that individual met inclusion criteria) and validating data from one individual, and not from two people who are in a dyadic relationship with one another (eg, same-sex male couple, mother, and daughter). Although many of the same data validation and screening recommendations for Web-based studies with individual participants can be used for those with dyads, there are differences and challenges that need to be considered. In particular, verification must be expanded beyond the individual-level, such that eligibility screener data must be collected from both participants of the dyad to compare and assess whether the two individuals represent a dyad (or not). As noted in a previous study, it is recommended for researchers to use predetermined decision rules regarding what response ranges will be acceptable per dyad when comparing one member’s answer with the other member’s answer [7]. Similar recommendations for validity data checking, as described above, can be applied to Web-based studies with dyads, yet other considerations must be made with respect to back-to-back data entries and multiple entries originating from the same IP address. For instance, some dyads may have both members using the same IP address and/or have one member refer the other to participate, resulting in back-to-back data entries for the dyad. As such, Web-based research studies with dyads may require different parameters or decision rules for validating dyadic data.

### Goal of the Study

With the overarching goal of improving Web-based verification and validation of couples’ relationships and associated data, we conducted a descriptive evaluation of the procedures used in a Web-based study with same-sex male couples. Specifically, this paper will describe the methods we used to verify and validate couples’ relationships and data (ie, whether two partners were in a relationship together as a couple, detection of fraudulent cases). The lessons we learned from this project can then be applied to future Web-based studies that involve screening and enrollment of couples with the collection of dyadic data.

## Methods

### Procedure Overview

**Figure 1:**
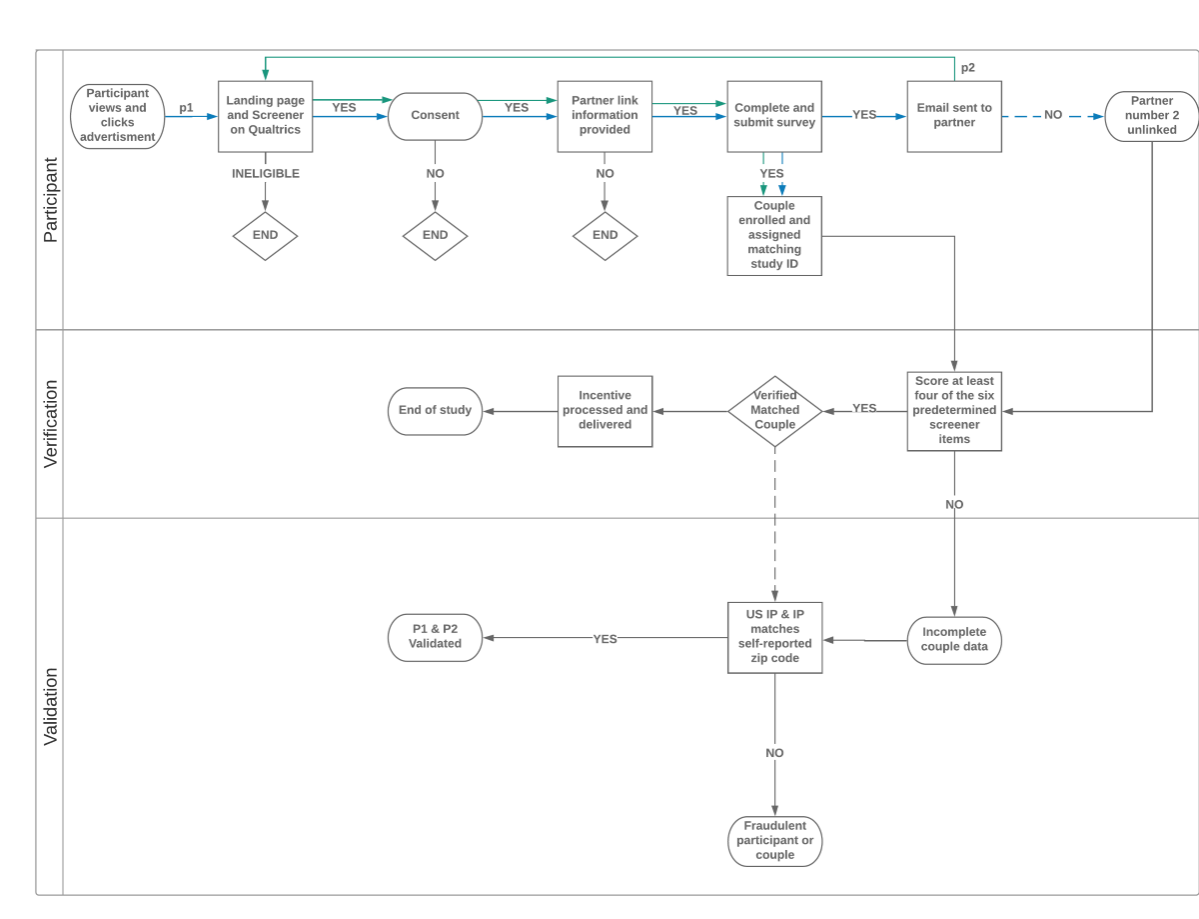
Overview of screening and enrollment procedures used. IP: internet protocol; p1: partner 1 ; p2: partner 2.

The University of Michigan Institutional Review Board (Protocol number HUM00125711) approved all study procedures. To accomplish the overarching goal of this study, we used data from a mixed methods study conducted about how factors shape partnered men’s support and willingness to use pre-exposure prophylaxis among concordant HIV-negative and HIV-discordant same-sex male couples in the United States. A variety of social media platforms (eg, Facebook, Instagram, Scruff) were used to target and recruit the convenience study sample. Figure 1 illustrates the enrollment procedures used for this study. Specifically, interested men who clicked on a social media advertisement were taken to the study landing webpage that briefly described the study and linked them to the eligibility screener before proceeding to the consent webpage. After providing consent, potential participants were then asked to provide their own and their partners’ contact information before accessing the online, cross-sectional study survey; we refer to this participant as the index partner of the couple. At this point in time, the partner of the index partner (ie, partner number 2) would then receive an email invitation containing a weblink with an embedded linkage code to the study landing webpage that would allow the individual to follow the same procedures for eligibility, consent, and accessing the survey as the index partner (green line in Figure 1). The linkage code and screener items were used to help match and then verify whether the two partners were a couple. To enroll into the study as participants, both partners of each couple had to meet all the eligibility criteria, consent, complete the study survey, and pass the verification and validation benchmarks. Each individual who completed the study survey was provided with an incentive (US $50 Amazon gift card), irrespective of his partner’s participation.

### Eligibility Criteria

Through self-reports, both partners of the couple had to meet the following study eligibility criteria: (1) self-identify as a cisgender male, (2) be 18 years of age or older, (3) live in the United States, (4) be in a sexual and romantic relationship with each other for 3 or more months, (5) engage in condomless anal sex at least once with their partner in the 3 months before assessment, and (6) both be HIV negative or be HIV sero-discordant.

### Procedures Used to Verify Couples’ Relationship and Validate Their Data

On the basis of our previous experiences of conducting Web-based studies with same-sex male couples [7], we developed and employed a process to help verify whether both partners were a true couple, as well as whether valid data were collected from each respective partner of the couple (Figure 1). The aim was to prevent individuals registering twice as a fake couple, or two people who were not in a relationship registering as a couple. For example, a feature we used in the eligibility screener on Qualtrics (SAP) was the *prevent ballot box stuffing*, which helps prevent someone from taking the same survey multiple times by attaching a cookie to their web browser to produce a message stating they had already taken the survey if they tried to take it again. *Verification of a couple’s relationship* was based on participants’ responses to six screener items with predetermined decision rules and the degree to which the couple had both partners concur on these items or reported responses within an acceptable range. As shown in Table 1, some items used to verify a couple’s relationship required that both partners of the couple report the same response, whereas other items allowed a reasonable margin of error (eg, within 1 year of age) to account for the potential of normalcy of human error as well as real life occurrences between the times of when each partner completes the screener (eg, possibility of a birthday happening). Six screener items with corresponding decision rules (ie, benchmarks) were used to verify the couple’s relationship.

Verification of each couple’s relationship was conducted manually by downloading data from Qualtrics, comparing both partner’s responses with these six items, and assigning the couple a score (range 0-6) based on the number of items passed following the predetermined decision rules (eg, 5 out of 6). Two team members compared the dyadic data and initially assigned the couples a verification score. A third team member then cross-checked the work conducted by the two team members. Data verification took 10 to 15 minutes per couple. Couples with a score of 4 or higher were considered as being in a relationship, whereas those who received a score of 3 or lower did not pass this benchmark and were marked as unverified. The use of a conservative score of 4 as the minimum to verify a couple’s relationship was based on balancing between the possibility for recall bias and human error, in recognizing that some partners may have multiple email addresses, not accurately recall when they last had condomless anal sex, or may have different initials from the name(s) one prefers or is often called (eg, John Paul Maxlin, goes by JP yet has first and last name initials of JM).

Once a couple passed the relationship verification benchmark, the *validity of their data* was assessed to determine that responses came from two unique individuals in a relationship and not from one person pretending to be two people (ie, fraudulent). Our validity test consisted of an evaluation of four criteria: US-based IP address (yes or no), IP address matched self-report of zip code (yes or no), number of data entries from the same IP address (3 or less), and start and stop times of each partner’s survey response. In addition to requiring the first two items passing the criteria (ie, both yes), no more than three entries were permitted to occur from the same IP address to allow for the possibility of fluxes in internet connectivity and both partners using the same internet connection. Back-to-back survey entries (ie, one survey completed, then second survey started shortly after) were permitted as long as the other three validation criteria passed. Overall, each couple had to pass the first two validation criteria and have no more than three screener entries between the two partners. Couples which passed the relationship verification test but failed the validation were deemed as fraudulent.

**Table 1:** Screener items with accompanying decision rules used for couple verification test.

Item	Relationship verification rules for responses	
	Partner 1 (index)	Partner 2
1. Partner 1 age	N/A^a^	± 1 year
1. Partner 2 age	± 1 year	N/A
2. Partner 1 birthday month	N/A	Exact
2. Partner 2 birthday month	Exact	N/A
3. Relationship length	Same response	Same response
4. Recently had condomless anal sex with partner	Same response	Same response
5. Partner 1 initials of first and last name	N/A	Exact
5. Partner 2 initials of first and last name	Exact	N/A
6. Partner 1 email/cell number	N/A	Must match one
6. Partner 2 email/cell number	Must match one	N/A

^a^N/A: not applicable.

### Analyses

Descriptive statistics (counts, proportions) were calculated to describe the sample relative to the verification and validation procedures. Comparative analyses via chi-square tests were used to assess whether any significantly meaningful demographic differences existed by couples’ verification score among those who had a benchmark of 4 and higher (ie, 4 vs 5 vs 6). Analyses were conducted using STATA version 15.

## Results

### Eligibility

**Figure 2:**
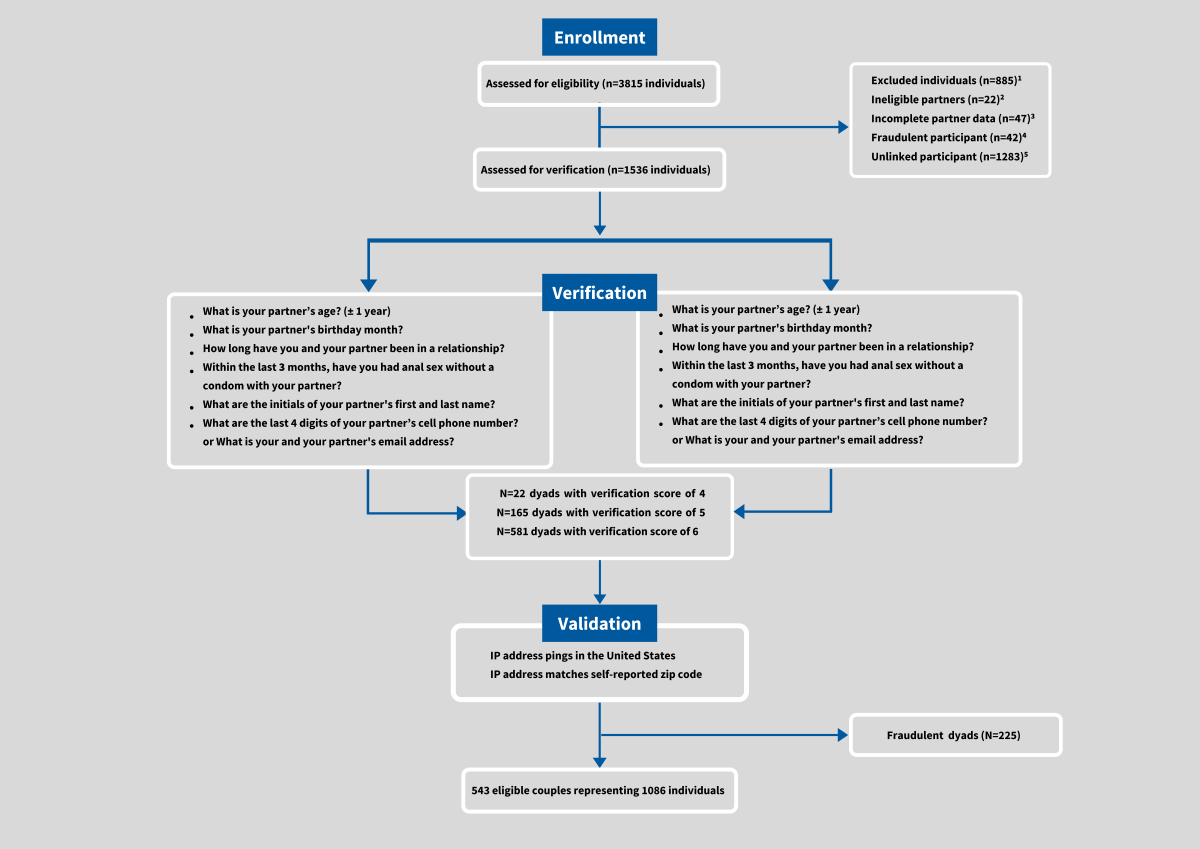
Consolidated Standards of Reporting Trials diagram of couple verification and validation procedures for enrollment. IP: internet protocol.

As shown in Figure 2, 3815 individuals were assessed for eligibility. Of these 3815 individuals, 2279 were excluded and the remaining 1536 individuals were matched with a corresponding partner (768 dyads) and evaluated for the relationship verification test. Of the 2279 who were excluded, the primary reasons were having an unlinked partner (n=1283; only partner A provided contact information) and partner A not completing all the questions on the screener (n=885). Others were excluded for having ineligible partners (n=22), incomplete partner data (n=47), and being detected as a fraudulent participant (n=42). The primary reasons detected for fraud were not living in the United States and/or having a fictitious identity. All 768 dyads passed the verification test by receiving at least a minimum score of 4 out of 6 screening rule items.

### Relationship Verification and Data Validation

Our descriptive analysis of the verification rules revealed approximately 2.9% (22/768) of dyads received a score of 4, 21.5% (165/768) of dyads received a score of 5, and 75.7% (581/768) of dyads received a score of 6. Some items used for the verification screening were missed more than others (Table 2) and tended to vary by couples’ verification score. A higher proportion of dyads with a score of 4 failed to pass the verification test for any given item, except their email addresses. Some dyads with a verification score of 4 or 5 had responses that did not match for their partner’s first and last name initials, relationship length, or both of these criteria. Interestingly, a similar proportion of dyads with a verification score of 4 or 5 had partners whose responses about their recent engagement in condomless anal sex in the relationship did not match.

**Table 2:** Proportion and identification of eligibility screening items that did not pass the verification test, by couples’ passing verification score.

Item	Couple verification score		
	4 (n=22), n (%)	5 (n=165), n (%)	6 (n=581), n (%)
Partner’s initials	19 (86.4)	86 (52.1)	0 (0.0)
Partner’s age	1 (9.0)	3 (1.8)	0 (0.0)
Partner’s birthday month	3 (13.6)	10 (6.0)	0 (0.0)
Relationship length	17 (77.3)	93 (56.4)	0 (0.0)
Recent condomless anal sex with partner	3 (13.6)	18 (10.9)	0 (0.0)
Partner’s cell numbers	4 (19.2)	13 (7.9)	17 (2.9)
Partner’s email addresses	0 (0.0)	32 (19.4)	24 (4.1)

For validation, 225 of the 768 dyads (29.3%) did not pass our test and were considered fraudulent. The 225 dyads did not pass the data validity test because one or both ‘partners’ used an IP address located outside of the United States and/or the IP address did not match the zip code self-reported in the survey. Overall, a total of 543 couples (consisting of 1086 partnered men) passed our verification and validation procedures and were enrolled into the study as participants.

To help improve screening procedures for verification of a couple’s relationship, we also explored whether demographic differences comparatively differed by a couple’s passing verification score (Table 3). Relationship length significantly differed between the three groups of couples according to their passing verification scores (4 vs 5 vs 6; *P*<.001. No other demographic characteristic significantly differed when comparing couples by their passing verification score.

**Table 3:** Descriptive statistics for participant demographics, by couples’ verification score (CVS).

Demographic		Total (n=1086), n (%)	CVS=4 (n=44), n (%)	CVS=5 (n=324), n (%)	CVS=6 (n=718), n (%)		*P* value
**Race/ethnicity**						.13	
	Non-Hispanic white	811 (74.68)	28 (63.64)	242 (74.69)	541 (75.35)		
	White/Hispanic	76 (7.00)	4 (9.09)	23 (7.10)	49 (6.82)		
	Black/Latino	57 (5.25)	7 (15.91)	12 (3.70)	38 (5.29)		
	Hispanic/Latino	47 (4.33)	2 (4.55)	18 (5.56)	27 (3.76)		
	Asian	34 (3.13)	2 (4.55)	11 (3.40)	21 (2.92)		
	Other^a^	61 (5.62)	1 (2.27)	18 (5.56)	42 (5.85)		
**Age (years)**						.58	
	18-24	160 (14.73)	3 (6.82)	46 (14.20)	111 (15.46)		
	25-34	637 (58.66)	28 (63.64)	191 (58.95)	418 (58.22)		
	35-44	217 (19.98)	9 (20.45)	61 (18.83)	147 (20.47)		
	45+	72 (6.63)	4 (9.09)	26 (8.02)	42 (5.95)		
**Region**						.50	
	Northeast	186 ‏(17.22)	10‏ (22.73)	51 ‏(15.74)	125‏ (17.56)		
	South	333 ‏(30.83)	14‏ (31.82)	102 ‏(31.48)	217‏ (30.48)		
	West	223 ‏(20.65)	12 ‏(27.27)	63‏ (19.44)	148 ‏(20.79)		
	Midwest	338‏ (31.30)	8 ‏(18.18)	108 ‏(33.33)	222‏ (31.18)		
**Education^b^**						.08	
	Up to high school graduate or equivalent	77 (7.12)	3 (6.81)	27 (7.39)	47 (7.05)		
	Some college education or technical school graduate	245 (22.65)	18 (40.91)	63 (26.52)	164 (21.91)		
	College graduate	378 (34.94)	12 (27.27)	115 (31.30)	251 (35.64)		
	Some graduate school or degree	382 (35.31)	11 (20.45)	118 (34.78)	253 (62.13)		
**Employment**						.78	
	Work full-time (30+ hours)	863 (79.76)	35 (79.55)	251 (77.71)	577 (80.70)		
	Work part-time (1–29 hours)	122 (11.28)	4 (0.09)	40 (12.38)	78 (10.91)		
	Unemployed/retired	97 (8.97)	5 (11.36)	32 (9.91)	60 (8.39)		
**Housing status**						.72	
	My own house or apartment	882 (81.52)	37 (84.09)	270 (83.59)	575 (80.42)		
	In my significant other’s house or apartment	106 (9.80)	2 (4.55)	30 (9.29)	74 (10.35)		
	At my parent’s house or apartment	44 (4.07)	2 (4.55)	10 (3.10)	32 (4.48)		
	Other^c^	50 (4.61)	3 (6.82)	14 (4.03)	34 (4.76)		
**Relationship type**						.08	
	Boyfriend/lover	416 (38.45)	13 (29.55)	122 (37.77)	281 (39.30)		
	Partner	232 (21.44)	7 (15.91)	64 (19.81)	161 (22.52)		
	Husband/spouse	404 (37.34)	24 (54.55)	131 (40.56)	249 (34.83)		
	Other^d^	30 (2.77)	—	6 (1.86)	24 (3.36)		
**Relationship length**						<.001	
	More than 3 months but less than 1 year	132 (12.15)	4 (9.09)	46 (14.20)	82 (11.42)		
	1 year but less than 3 years	348 (32.04)	12 (27.27)	85 (26.23)	251 (34.96)		
	3 years but less than 5 years	231 (21.27)	17 (38.64)	63 (19.44)	151 (21.03)		
	More than 5 years	375 (34.53)	11 (25.00)	130 (40.12)	234 (32.59)		

^a^Includes 5 Native American/Alaskan Native, 5 Native Hawaiian/Other Pacific Islander, 1 Indian, 1 Middle Eastern, 1 Caribbean, and 48 mixed.

^b^For Education, Employment, Housing status, Relationship type, and Relationship length, the sample size is 1082 for total, 44 for CVS=4, 323 for CVS=5, and 715 for CVS=6.

^c^Includes college dorm, employee housing, sharing with significant other.

^d^Includes fiancé, mates, interchanging use of partner, boyfriend, or husband.

## Discussion

### Principal Findings

Several lessons were learned from the descriptive evaluation we conducted on the verification and validation procedures used to screen and enroll same-sex male couples in this Web-based study. First, some of the screening items used in the verification test were missed more than others, suggesting the need to consider either amending these items or to use entirely different items to verify a couple’s relationship. The measure used for relationship length contained overlapping categorical response options (eg, 3-6 months, 6-12 months) that may help explain why some partners of couples had reported different responses. It is also possible that partner’s definition of *when* their relationship began may have differed from one another. To help prevent the potential for measurement and interpretation error, we recommend improving the response options for this item by: 1) eliminating any overlap of time between each potential response and 2) using a suggested *event* as a potential start date of the couple’s relationship (eg, first date, decided to be in a relationship with one another). However, this item alone will not account for the possibility for recall bias or that some couples may have broken up for a short period of time and had gotten back together, suggesting the potential for partners to still report different timeframe responses for their relationship length, depending on when they consider the start or restart of their relationship. Thus, we recommend adding an additional screening item to the verification procedure to assess whether the couple had previously broken up or taken a break in their relationship (yes or no), in addition to asking about their relationship length. In sum, these suggestions may help with future assessment of a couple’s relationship length and the degree to which partners concur about their relationship length as an item to include in a verification test.

The other verification item missed by a substantial proportion of couples was partner’s initials for their first and last name. In our analysis of the data, we noticed two trends that may help explain why some couples did not pass this item. Some participants may have mistyped and entered the incorrect letter either for their own or partners’ initials. Other participants reported more than two initials for their own and/or partners’ name, whereas their partner reported exactly two initials. It is possible that a participant’s name may have more than two initials, such as having a middle name or two first names (eg, John Paul), as well as preferring to be called and known by their middle name instead of their first name given at birth (eg, Xavier Michael, goes by Michael). Given the variability between actual, known, and preferred name, we recommend replacing this verification item and using a simpler one (eg, cohabitation, presence of tattoos) with a categorical response (eg, yes or no) for future Web-based studies with couples. Thus, verification items which contain responses with concrete interpretation may help reduce the chances for human error although they also increase ease of interpretation for the participant. Future research that uses qualitative methods to explore couples’ thoughts and suggestions for what questions researchers could use to verify their relationship in Web-based studies is needed. For instance, couples could assist with identifying new topics (eg, pet ownership), as well as with the creation of new screening items with accompanying decision rules, thereby updating and potentially improving the verification process with their input.

Next, a large amount of resources (eg, personnel, time) were needed to apply the verification and validation tests. Both tests were manually checked for a total of three times, with each check being done by a different, independent member of the research team. Discrepancies were resolved through discussion, referring back to the predetermined decision rules (eg, exact response required) and reaching a consensus. No human errors were found for the validation tests, but several errors were found for the verification tests. Although human error will always remain a possibility when cross-referencing and comparing data responses, we recommend that future work consider for this possibility by allocating appropriate time and personnel. As noted by a previous Web-based study with couples [7], another option for researchers to consider is the creation and use of an electronic algorithm that automatically compares partners’ responses with the eligibility screener for the relationship verification test. At present, it is unclear whether the manual check or electronic algorithm option would be more cost and time effective to conduct to verify whether both partners of the couple or dyad are in a relationship with one another (ie, verification test). Future research is needed in this area to assess and compare which approach (ie, manual vs electronic algorithm) would be more time and/or cost efficient, while accounting for variability in a study’s sample size (eg, 50 couples vs 500 couples).

Third, the email invitation containing the weblink with an embedded linkage code was not 100% reliable to exclusively link both partners together as a couple. To recap, the email with the embedded code was sent to partner 2 once index partner provided consent and entered partner 2’s contact information. Some partners (ie, partner 2’s) independently completed the screener not using the email invitation containing the embedded linkage code. As we required contact information from each participant for both partners of the couple, we were still able to link partners together as a couple by cross-referencing to see whether their emails and/or mobile phone numbers matched. Although we still recommend using an email invitation delivery system with an embedded linkage code for the index partner to refer their partner to participate, we also highly recommend for researchers to require each partner to input his own and his partner’s contact information as an additional safeguard. This two-pronged mechanism will help researchers identify any potential mismatched partners of couples when implementing the verification and validation procedures. In other words, the phone numbers and email addresses can be used to cross-reference to help find pairs of partners as potential couples.

Finally, the order in which we applied the verification and validation tests (ie, procedures) for this study did not account for the possibility of when fraudulent cases could flood the eligibility screener (eg, *bots*) database system, and how best to handle when these instances occur. For this study, we applied the verification test before the validation test for each couple. Toward the end of recruitment, we received over 400 entries in the study eligibility screener in a relatively short period of time; all of these data entries passed the verification test perfectly (6/6) yet failed the validation test and were labeled as fraudulent data. Evaluating these fictitious data entries was time consuming and yet, had we not implemented this step, approximately 30% of fraudulent couples would have been included in the study and would have impacted the overall findings. For future Web-based studies that seek to enroll data from both partners of a couple, we recommend for researchers to monitor data entries for the eligibility screener on a daily basis (if possible) to note if and when any patterns emerge during recruitment. In our case, we noted that hundreds of odd email address handles (eg, jlqdpz7dm2@live.com) and/or highly similar phone numbers (eg, 888-123-3435, 888-123-3434) were imputed for each given dyad, along with back-to-back screener entries (ie, consecutive start and stop times). Further, these fraudulent data entries occurred in a relatively small period of time (eg, 24 hours), adding to the suspicion that the data were invalid. Inserting captchas—a mechanism that requires an individual to recognize and identify a certain object within a larger image—at the beginning of a survey could provide researchers with a good option to help deter bot survey responses. In addition, if an electronic algorithm method is used, then safeguards could be implemented to help block and prevent instances of when large volumes of *bots* and other forms of fictitious data flood an eligibility screener database. Specifically, an electronic algorithm method could enable researchers to set parameters about the number of eligibility screener entries to permit per IP address, requiring the IP address to be US-based, and whether a minimum amount of time is needed between the stop time of one partner’s data entry relative to the start time of the second partner’s data entry (ie, back-to-back). These suggestions may help block *bots* and other forms of fictitious data from flooding an eligibility screener database, which may be more likely to happen when a Web-based research study offers a participant an incentive. Other studies have reported such instances relative to fraudulent data entries [5,11-17], though none were with couples and dyadic data. As such, the use of an automated, electronic algorithm may serve as an additional advantage to help deter the receipt of large volumes of fraudulent and fictitious data entries during the enrollment process for Web-based research studies.

### Limitations

This study is not without limitations. First and foremost, all data come from a Web-based, convenience sample of same-sex male couples who may not be representative of other same-sex male couples in the United States (and elsewhere). Further, individuals who decided to complete the eligibility screener may be different from others, given the topic of the research study was about HIV prevention as opposed to another health topic, such as stress. The efficacy of the items used to verify couples’ relationships has also not been done and warrants future investigation with this population and other groups of couples. Nonetheless, the recommendations we provide based on the experiences of using the present verification and validation enrollment procedures are applicable to other Web-based studies which seek to enroll and collect dyadic data from couples.

### Conclusions

Findings from this descriptive evaluation draw from our experience of recruiting and enrolling a large sample of same-sex male couples into a Web-based HIV prevention study. The procedures we used to verify and validate that both the partners were in a relationship together and had independently provided data illuminated potential areas for improvement. We offer examples and considerations relative to improving screening items for the verification process, and a call for further research to compare the advantages and disadvantages of implementing such procedures manually versus electronically. Collectively, additional methodological research that aims to streamline the process of enrolling verifiable couples and collecting valid dyadic data is needed, as more and more research studies are conducted over the Web.

## Abbreviations

IP: internet protocol
